# Treatment of old landfill leachate with high ammonium content using aerobic granular sludge

**DOI:** 10.1186/s13036-017-0085-0

**Published:** 2017-11-09

**Authors:** Yanan Ren, Fernanda Ferraz, Abbass Jafari Kang, Qiuyan Yuan

**Affiliations:** 1 Department if Civil Engineering, University of Manitoba, 15 Gillson St., Winnipeg, MB R3T 5V6 Canada; 2 Department of Hydraulics and Sanitation, University of Sao Paulo, Av. Trabalhador Sao-Carlense, 400, Sao Carlos, SP 13566-590 Brazil

**Keywords:** Activated sludge, Aerobic granular sludge, Landfill leachate, Free-ammonia, Nutrients removal, Organic matter removal, Phosphorus removal, Sequencing batch reactors, Simultaneous nitrification-denitrification

## Abstract

**Background:**

Aerobic granular sludge has become an attractive alternative to the conventional activated sludge due to its high settling velocity, compact structure, and higher tolerance to toxic substances and adverse conditions. Aerobic granular sludge process has been studied intensively in the treatment of municipal and industrial wastewater. However, information on leachate treatment using aerobic granular sludge is very limited.

**Methods:**

This study investigated the treatment performance of old landfill leachate with different levels of ammonium using two aerobic sequencing batch reactors (SBR): an activated sludge SBR (ASBR) and a granular sludge SBR (GSBR). Aerobic granules were successfully developed using old leachate with low ammonium concentration (136 mg L^−1^ NH_4_^+^-N).

**Results:**

The GSBR obtained a stable chemical oxygen demand (COD) removal of 70% after 15 days of operation; while the ASBR required a start-up of at least 30 days and obtained unstable COD removal varying from 38 to 70%. Ammonium concentration was gradually increased in both reactors. Increasing influent ammonium concentration to 225 mg L^−1^ N, the GSBR removed 73 ± 8% of COD; while COD removal of the ASBR was 59 ± 9%. The GSBR was also more efficient than the ASBR for nitrogen removal. The granular sludge could adapt to the increasing concentrations of ammonium, achieving 95 ± 7% removal efficiency at a maximum influent concentration of 465 mg L^−1^ N. Ammonium removal of 96 ± 5% was obtained by the ASBR when it was fed with a maximum of 217 mg L^−1^ NH_4_^+^-N. However, the ASBR was partially inhibited by free-ammonia and nitrite accumulation rate increased up to 85%. Free-nitrous acid and the low biodegradability of organic carbon were likely the main factors affecting phosphorus removal.

**Conclusion:**

The results from this research suggested that aerobic granular sludge have advantage over activated sludge in leachate treatment.

## Background

Landfilling is used worldwide as a strategy for municipal solid waste (MSW) disposal. Modern landfills offer a safe final disposal of MSW; however, when the liquids (e.g. precipitation, water content of the waste, melted snow) seep through the waste, it generates a wastewater called leachate. Among its constituents, there are heavy metals, dissolved solids, ammonia, biodegradable, and refractory organic matter. To prevent impacts on the environment and human health, leachate must be collected from the landfills and treated. According to the biodegradation stage of MSW, the concentrations of these compounds may vary and leachate can be classified as old or young. The latter refers to leachates containing a high amount of volatile fatty acids (VFAs), whereas old leachates are mostly constituted by refractory organic matter (as humic substances) and high ammonia nitrogen concentrations [1]. Biological processes have been used worldwide to treat leachate. They were particularly effective for the treatment of young leachate containing easily biodegradable organic matter [1–3]. However, there are concerns about the application of biological processes for the old leachate treatment, which contains high concentrations of ammonium nitrogen and refractory organic matter [1]. Application of chemical treatments especially advanced oxidation processes (AOP) such as sulfate radical [4] and Fenton [5] were found effective to reduce the refractory organic content of old leachate. Despite the good results, chemical methods typically need to be combined to produce a treated effluent in accordance with restricted discharge limits. Consequently, its application may be limited by the high operational costs related to energy and chemicals consumption [1]. Certain strategies may be adopted upon the feasibility of the old leachate treatment by biological processes, including pre-treatments and co-treatment with domestic wastewater. Chemlal et al. [6] found that AOP as a pre-treatment could enhance the performance of an aerobic bioreactor by increasing the biodegradability of the refractory fractions. The AOP-bioreactor allowed an abatement of 90% of biological oxygen demand (BOD) and 87% of COD from an old leachate. According to Yuan et al. [7] pre-treatment of old leachate via air stripping followed by co-treatment with wastewater in an aerobic SBR (V_leachate_/V_wastewater_ = 2.5%) obtained 87% and 100% of COD and phosphorus removal, respectively. Similar results have been reported in other research works [8, 9]. However, increasing volumetric ratio of the air-stripped leachate (up to 50%) was found to influence nutrient removal in both aerobic and anaerobic co-treatment of leachate and wastewater [10].

Recent studies have discussed the treatment of different industrial wastewaters using aerobic granular sludge (AGS) and reported their higher tolerance to toxic substances and adverse conditions compared to the activated sludge process [11, 12]. However, information on leachate treatment using AGS is very limited. In a study which is the only one in this area, aerobic granular sludge obtained maximum removals of 83% and 92% for COD and ammonium from raw old leachate, respectively [13].

Considering that the literature is lacking information regarding leachate treatment by AGS, this study aimed to assess old leachate treatment by AGS in comparison with the activated sludge process. The study was performed using two SBR, an activated sludge SBR (ASBR) and an aerobic granular sludge SBR (GSBR). The reactors were evaluated based on the organic matter and nutrient removal efficiencies.

## Methods

### Experimental set-up

**Fig. 1:**
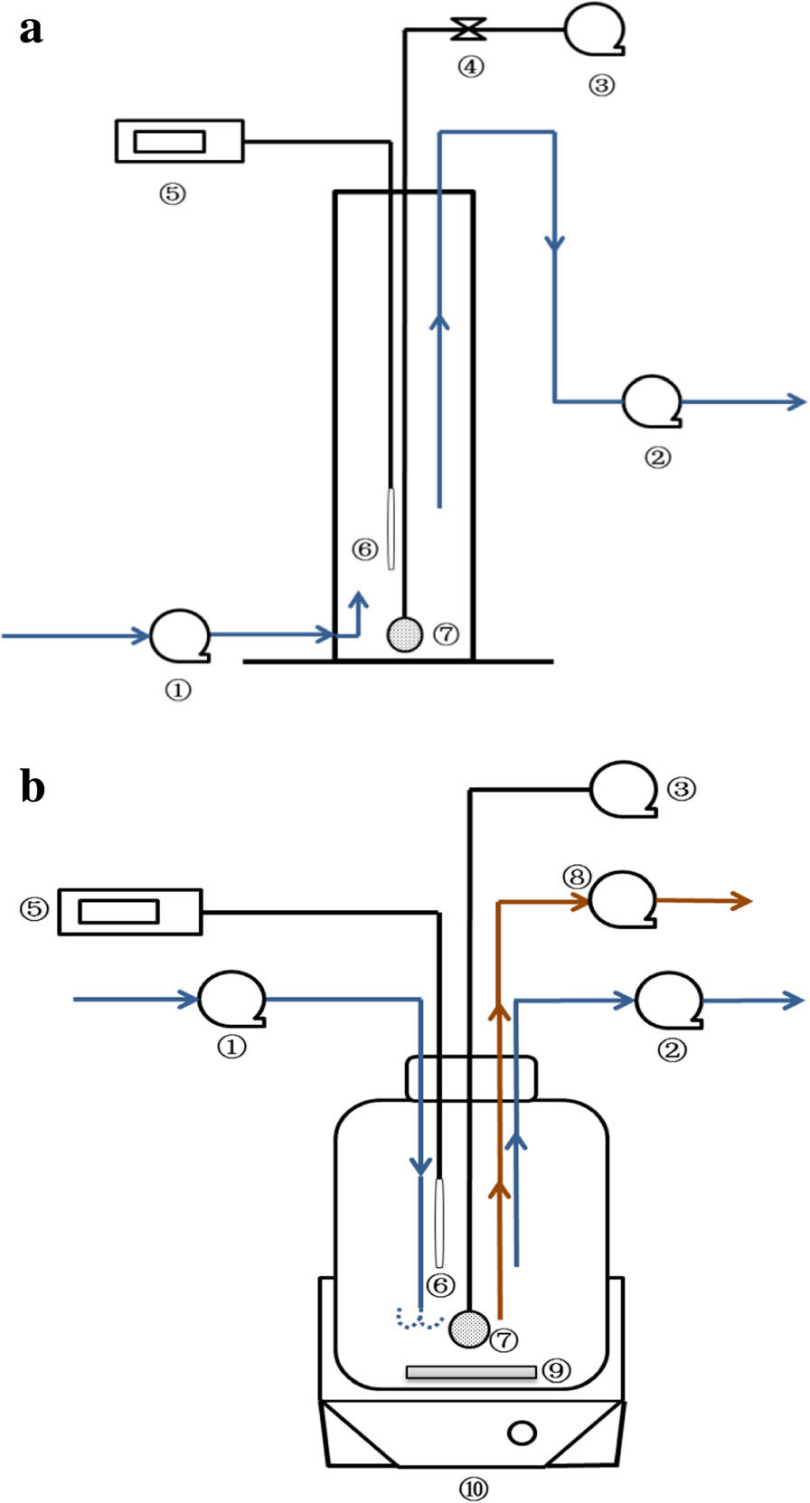
Schematic of the **a** GSBR and **b** ASBR: ① feeding pump; ② decanting pump; ③ air compressor; ④ air flow meter; ⑤ pH controller; ⑥ pH meter; ⑦ air diffuser; ⑧ waste sludge pump; ⑨ stir bar; ⑩ magnetic stir plate

The GSBR consisted of a plexiglass cylinder with a 12-cm internal diameter, the height of 45-cm and the total volume of 5 L, whereas the working volume was 3 L (Fig. 1a). The ASBR consisted of a 5 L glass jar, plexiglass cylinder having a 15-cm diameter and 30-cm height, whereas the working volume was 3 L (Fig. 1b). The reactors were inoculated with 1 L of activated sludge collected from a full-scale wastewater treatment plant (South End Water Pollution Control Centre in Winnipeg, MB, Canada). For both the ASBR and the GSBR, at each cycle 1.5 L of supernatant (treated effluent) was withdrawn from the reactors and 1.5 L of fresh feed was pumped into them, keeping a working volume of 3L and an exchange ratio of 50%.

The SBRs were provided with an up-flow feed from the bottom of the apparatus. Aeration was provided by a fine air diffuser and a flow meter controlled the airflow at 3 L min^−1^ resulting in superficial air velocity of 0.44 cm/s. The pH of both reactors was kept greater than 6.5 during the aerobic/anaerobic reaction time using sodium bicarbonate solution.

### Operation of reactors

The reactors were operated at 3 cycles per day (8 h each cycle), at room temperature (20 ± 2 °C). For the GSBR, each cycle consisted of a 30-min feeding period, 1.5-h anoxic/anaerobic period, 5.75-h aerobic period, 5-min settling period, 12-min decanting period and 3-min idle phase. For the ASBR, each cycle consisted of a 30-min feeding period, 1-h anoxic/anaerobic mixing period, 5.5-h aerobic period, 40-min settling period, 12-min decanting period, and 3-min idle phase. At the end of the aerobic phase, 30 ml of the ASBR mixed liquor was eliminated to obtain SRT of 30 days. However, sludge retention time (SRT) of granular sludge was not controlled and biomass was only wasted via effluent solids.

### Synthetic old landfill leachate wastewater

A synthetic old landfill leachate was prepared with tannic acid, which was used to simulate the refractory organic matter content of old leachates [14–16]. Additionally, macro and micronutrients were dissolved in tap water (Table 1). For every cycle, 20 L of fresh feed was prepared and stored in a cold chamber at 4 °C.

**Table 1:** Composition of synthetic old landfill leachate

Organic and Inorganic Compounds		Trace metal solution	
Components	Per litre	Components	Per litre
Tannic acid	200 mg	CoSO_4_·7H_2_O	150 mg
NaCl	2000 mg	H_3_BO_4_	50 mg
CaCl_2_	700 mg	ZnSO_4_·7H_2_O	50 mg
NaHCO_3_	2000 mg	CuSO_4_·5H_2_O	40 mg
NaOH	297 mg	MnSO_4_·7H_2_O	500 mg
K_2_HPO_4_	32.5 mg	(NH_4_)_6_Mo_7_O_24_·4H_2_O	50 mg
NH_4_Cl	120-500 mg	Al_2_(SO_4_)_3_·16H_2_O	30 mg
Trace metal	0.02 ml	NiSO_4_·6H_2_O	500 mg
		96% H_2_SO_4_	1 ml

The ammonium nitrogen concentration in the influents was gradually increased in accordance with the increasing dosages of NH_4_Cl in the feed of both reactors. The nitrogen loads varied from 0.42 ± 0.04 kg m^−3^ d^−1^ (139 ± 14 mg L^−1^) to 1.39 ± 0.14 kg m^−3^ d^−1^ (465 ± 46 mg L^−1^) for the GSBR and from 0.41 ± 0.03 kg m^−3^ d^−1^ (136 ± 9 mg L^−1^) to 0.65 ± 0.08 kg m^−3^d^−1^ (217 ± 26 mg L^−1^) for the ASBR.

### Sampling and analytical methods

Samples were taken regularly from influent and effluent, being filtered (0.45 μm) before the physico-chemical analyses. Dissolved phosphate, total ammoniacal nitrogen (TAN), nitrate, and nitrite concentrations were measured by flow-injection analysis using a Lachat Instrument QuikChem 8500. Soluble COD (SCOD) was measured by Hach kits. Mixed liquor suspended solids (MLSS), mixed liquor volatile suspended solids (MLVSS), and sludge volumetric index (SVI) were quantified at 5 and 30 min according to [17].

The biomass of both granular and activated sludge was characterized in terms of particle size, SVI, and extracellular polymeric substances (EPS). Sludge particle size was determined by a Malvern Mastersizer 2000 analyzer. EPS were extracted from sludge by NaOH and formaldehyde method [18]. Protein and polysaccharides concentrations were measured by the modified Lowry assay kit and phenol-sulfuric acid colorimetric method, respectively [19].

Free-ammonia (FA) concentration was calculated by the Eq. 1, whereas free-nitrous acid (FNA) concentration was determined using Eq. 2 [20]. Total nitrogen (TN) was determined by the summation of NH_4_-N, NO_2_-N, and NO_3_-N [21]. Full nitrification, nitrite accumulation rate (NAR), denitrification efficiency (DE), and simultaneous nitrification-denitrification (SND) were determined by the Eqs. 3-5 [13] and Eq. 6 [22], respectively. The overall efficiencies of organic matter and nutrients removal were determined by the Eq. 7 [21].

1 $$ \mathsf{FA}\left(\mathsf{mg}{\mathsf{L}}^{-\mathsf{1}}\right)=\mathsf{17}/{\mathsf{14}}^{\ast}\frac{{\mathsf{NH}}_{\mathsf{4}}^{+}\times {\mathsf{10}}^{\mathsf{pH}}}{\exp \left(6334/273+\mathrm{T}\right)+{\mathsf{10}}^{\mathsf{pH}}} $$

2 $$ \mathit{\mathsf{FNA}}\left(\mathit{\mathsf{mg}}{\mathit{\mathsf{L}}}^{-\mathsf{1}}\right)=\mathsf{46}/{\mathsf{14}}^{\ast}\frac{{\mathit{\mathsf{NO}}}_{\mathsf{2}}^{-}}{{\mathit{\mathsf{Ka}}}^{\ast }{\mathsf{10}}^{\mathit{\mathsf{pH}}}} $$

3 $$ \mathsf{FullNitrification}\ \left(\%\right)={\mathsf{100}}^{\ast}\left(\frac{{\mathsf{NO}}_{\mathsf{3effluent}}^{-}}{{{\mathsf{NH}}_{\mathsf{4}}^{+}}_{\mathsf{removed}}}\right) $$

4 $$ \mathit{\mathsf{NAR}}\left(\%\right)={\mathsf{100}}^{\ast}\left(\frac{{\mathit{\mathsf{NO}}}_{\mathsf{2}\ \mathit{\mathsf{effluent}}}^{-}}{{\mathit{\mathsf{NO}}}_{\mathit{\mathsf{x}}\ \mathit{\mathsf{effluent}}}^{-}}\right) $$

5 $$ \mathit{\mathsf{DE}}\ \left(\%\right)={\mathsf{100}}^{\ast}\left(\mathsf{1}-\frac{{\mathsf{NO}}_{\mathsf{x}}^{-}\mathsf{accumulated}}{{\mathsf{TN}}_{\mathsf{Influent}}-{\mathsf{TN}}_{\mathsf{effluent}}}\right) $$

6 $$ \mathit{\mathsf{SND}}\ \left(\%\right)={\mathsf{100}}^{\ast}\left(\mathsf{1}-\frac{{\mathsf{NO}}_{\mathsf{x}}^{-}\mathsf{accumulated}}{{{\mathit{\mathsf{NH}}}_{\mathsf{4}}^{+}}_{\mathsf{removed}}}\right) $$

7 $$ \mathit{\mathsf{Efficiency}}\left(\%\right)={\mathsf{100}}^{\ast}\left(\frac{{\mathsf{C}}_{\mathsf{influent}}-{\mathsf{C}}_{\mathsf{effluent}}}{{\mathsf{C}}_{\mathsf{Influent}}}\right) $$

where C represents the concentration of COD, TN, NH_4_-N, or phosphorus.

### Statistical analysis

An analysis of variance (ANOVA) was performed to compare the performances of the aerobic reactors treating old leachate.

## Results and discussions

### Biomass characteristics

The GSBR and the ASBR were operated for 200 days. The ASBR was inoculated with 3430 mg L^−1^ of MLSS, but this value decreased to 1550 mg L^−1^ during a start-up period of 30 days. The activated sludge presented good settling properties: the values of SVI_5_ and SVI_30_ were 45 ± 1.8 ml g^−1^ and 42 ± 8 ml g^−1^, respectively. According to Metcalf and Eddy [23], good settleability was observed when the sludge presented SVI_30_ values smaller than 150 ml g^−1^.

The GSBR was inoculated with activated sludge at MLSS concentration of 4030 mg L^−1^. As granulation was developed, the MLSS concentration in the GSBR was gradually increased, reaching an average of 8070 ± 615 mg L^−1^ after 100 days. This value was maintained along with time, whereas the MLVSS represented about 86% of the MLSS concentration. The average size of the activated sludge was 118 μm and it increased up to 307 μm in approximately three weeks as the AGS was developed. Great settling properties were observed as the AGS was formed: the SVI_5_decreased from the initial value of 45 ± 1.8 ml g^−1^ to 25 ± 3.5 ml g^−1^.

Regarding EPS analysis, protein concentration (PN) in both reactors was quite similar: 30.75 mg g^−1^ VSS for the GSBR and 33.59 mg g^−1^ VSS for the ASBR (Table 2). However, the difference between the two types of sludge was highlighted by the polysaccharide (PS) concentration. Due to the intrinsic characteristics of granulation, i.e., granules backbone being constituted by PS [18], the PS concentration was higher in the GSBR than in the ASBR. The ratio PN/PS was on average 0.60 for the GSBR and 0.75 for the ASBR. The preponderance of PS over PN is associated with the hydrophilic properties of microbial aggregates [24].

**Table 2:** The average PN, PS concentration, and their ratio in GSBR and ASBR

Biomass	PN	PS	PN/PS
	(mg L^−1^)	(mg L^−1^)	
Aerobic granular sludge	30.80	70.13	0.60
Activated sludge	33.60	49.55	0.75

### COD removal

**Fig. 2:**
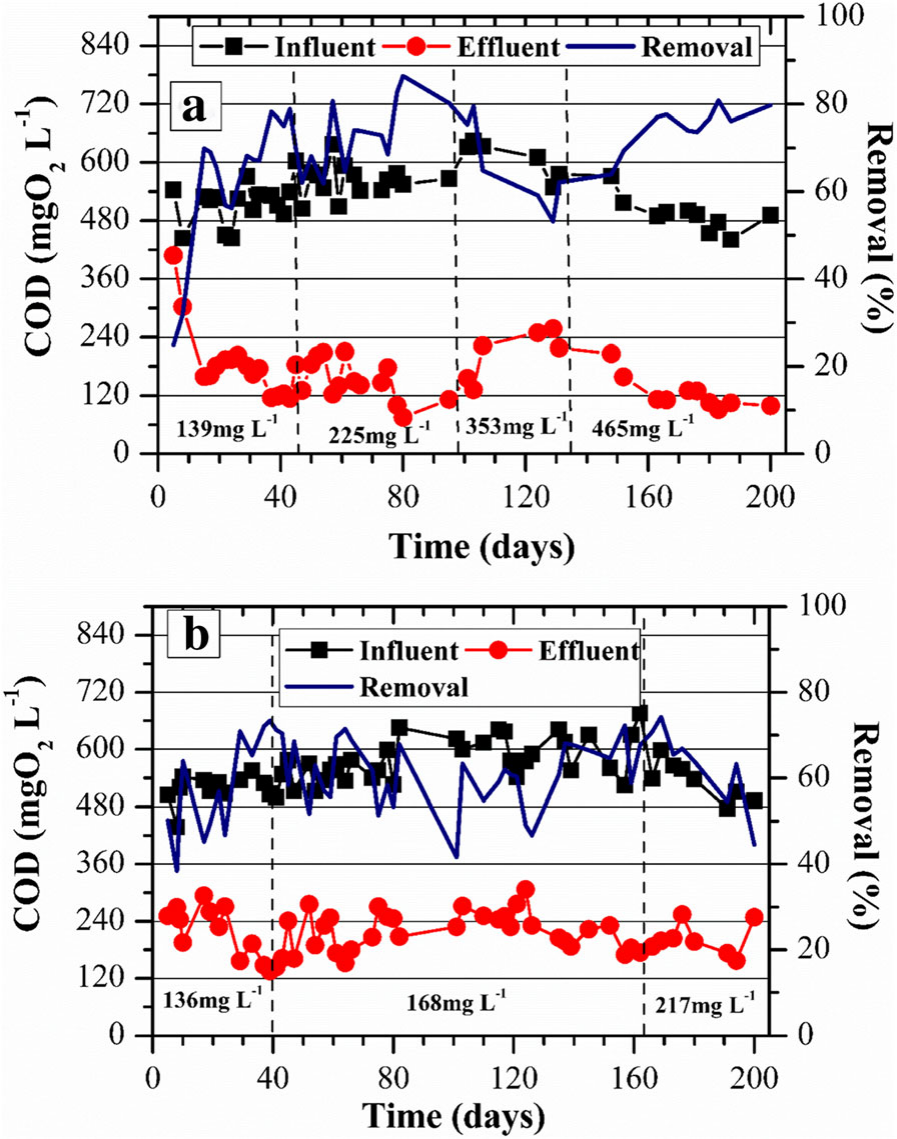
COD removal profiles along with time for the aerobic reactors: **a** GSBR and **b** ASBR. * The concentrations in the graphs refer to ammonium nitrogen

The influent COD concentrations were similar in both reactors, ranging from 448 to 654 mg L^−1^. As shown in Fig. 2, the GSBR was much more efficient in removing the COD rather than the ASBR. In other words, the granular sludge could tolerate higher concentrations of influent COD and ammonium nitrogen than those applied to the ASBR. As shown in Fig. 2a, after 15 days the GSBR was already removing 70% of COD, maintaining the stable removal efficiency in 73 ± 8% while the influent ammonium nitrogen was up to 225 ± 21 mg L^−1^. When the influent ammonium nitrogen concentration was increased to 450 mg L^−1^, COD removal decreased to 55 ± 8%. However, the granular sludge recovered its performance in maintaining a COD removal efficiency of 66 ± 12%. It was clear that the GSBR could tolerate a high ammonium nitrogen concentration of 465 ± 46 mg L^−1^. According to the ANOVA results, the differences among the GSBR removal efficiencies were statistically significant (*P*-value = 0.02 at α = 0.05).

In contrast to the GSBR, the ASBR did not present a stable COD removal during the first 30 days of the start-up where the efficiencies varied from 38 to 70% (Fig. 2b). While the influent ammonium nitrogen concentration was 136 mg L^−1^, COD removal was 59 ± 12%. As the ammonium nitrogen was slightly increased to 168 mg L^−1^, the biomass could tolerate such concentration and COD removal was slightly increased to 62 ± 8%. At the last stage of the experiment, where ammonium nitrogen was increased to 217 mg L^−1^, COD removal slightly decreased to 59 ± 9%. According to the ANOVA results, the differences among the ASBR removal efficiencies were random (*P*-value = 0.66 at α = 0.05); therefore, its performance resulted in a COD removal of about 60% for an influent ammonium nitrogen up to 217 mg L^−1^.

For the same range of influent ammonium concentration (up to 200 mg L^−1^), the ANOVA results showed that the different performances of the GSBR and the ASBR regarding COD removal were statistically significant (*P*-value = 0.02 at α = 0.05).

For an influent ammonium nitrogen concentration in the range of 200 mg L^−1^, the ANOVA results showed that the different performances exhibited by the two reactors regarding COD removal were statistically significant (P-value = 1.4 × 10^−6^ at α = 0.05).

### Nutrients removal

#### Nitrogen removal

**Fig. 3:**
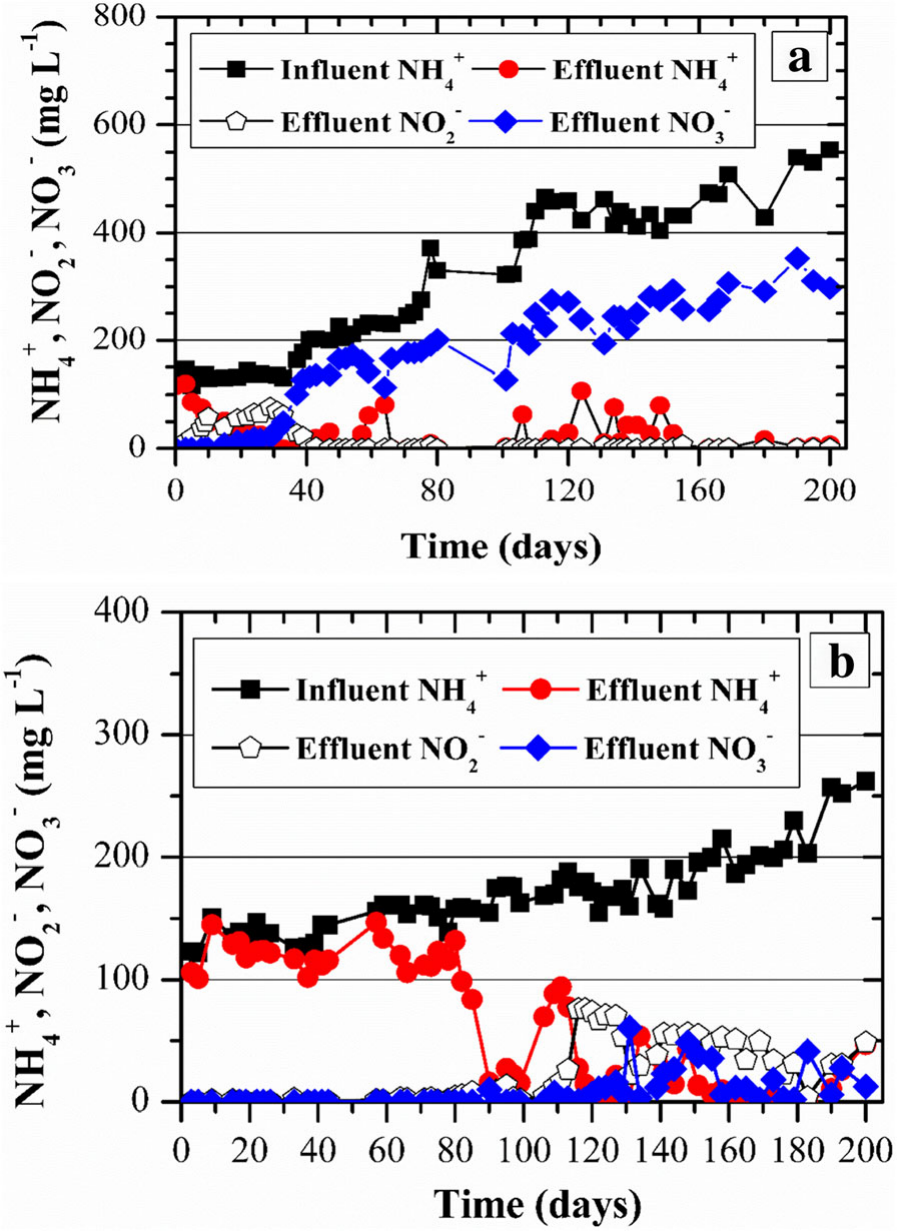
Ammonium nitrogen, nitrite nitrogen and nitrate nitrogen profile along with time: **a** GSBR and **b** ASBR

**Fig. 4:**
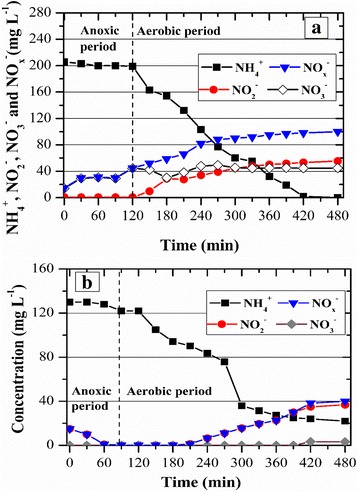
Nitrogen removal during a typical cycle by **a** the GSBR and **b** the ASBR

Results from measures of nitrogen concentration in the reactors influent and effluent are presented in Fig. 3. After 15 days, ammonium nitrogen removal (nitritation + nitrification) (Eq. 7) by the GSBR was already 61% (Fig. 3a). As the biomass became adapted to the increasing influent concentrations of ammonium nitrogen, the removal efficiencies were also increased. A maximum average of ammonium removal (nitritation + nitrification) of 95 ± 7% was obtained when the GSBR was loaded with 1.39 ± 0.14 kg m^−3^ d^−1^ (465 ± 46 mg L^−1^). The same trend was observed for TN, whereas the maximum removal efficiency of 39 ± 7% was observed when the maximum nitrogen load was applied. Accordingly, SND efficiency (Eq. 6) at the last stage of the experiment was 40 ± 7%, indicating that SND was most likely the primary mechanism of TN removal, which was further assessed by kinetic tests. Fig. 3a shows that production of nitrate increased with time and its concentrations were much higher than nitrite. At the last stage of the experiment, the full nitrification efficiency was 62 ± 9% (Eq. 3), suggesting that nitrifiers in the GSBR were not significantly inhibited by the lower influent FA concentration of 2.7 mg L^−1^ at pH of 7. This result was in agreement with the previous study that nitrifiers from activated sludge were inhibited at FA concentrations of 4 mg L^−1^ [25]. However, denitrification or SND do not seem to have been fully transpired, which was further assessed during a kinetic test (Fig. 4a).

According to a kinetic test performed on day 95 (Fig. 4a), the GSBR cycle started with 221 mg L^−1^ of TN and 121 mg L^−1^ were removed at the end of it (i.e., TN removal of 55%). The denitrification was only observed during the anoxic period. It is worth noting that despite the availability of carbon source during the anoxic period due to feeding, the incoming influent could not have been properly mixed with all the settled biomass, which resulted in the absence of significant denitrification activity. Additionally, tannic acid could have been less susceptible to biodegradation under anaerobic/anoxic conditions rather than under aerobic conditions, which was also observed by [26]. In contrast, during the aerobic period, 121 mg L^−1^ of TN were removed via SND at an efficiency of 59%. Our results were in agreement with other studies in the literature related to the treatment of ammonium-rich wastewaters [13, 27].

During the first 40 days, ammonium nitrogen removal (Eq. 7) by the ASBR was extremely low, 13 ± 6% (Fig. 3b), i.e., it was almost 7 times smaller than the efficiency presented by the GSBR for the same influent ammonium concentration. As the biomass became adapted to the increasing influent concentrations of ammonium nitrogen, the removal efficiencies were also increased. A maximum average of ammonium removal (nitritation + nitrification), 96 ± 5%, was obtained when the ASBR was loaded with 0.65 ± 0.08 kg m^−3^ d^−1^ (217 ± 26 mg L^−1^). The TN removal efficiencies (Eq. 7) also increased with time, reaching a maximum of 72 ± 10% when the maximum nitrogen load was applied. Nitrate was detected at concentrations as low as 10 mg L^−1^ only after 100 days, whereas during most of the experimental duration NAR (Eq. 4) ranged from 70 to 85% (Fig. 3b). A kinetic test was performed to better investigate the mechanisms for TN removal. In contrast to the GSBR, the influent FA concentrations (pH = 7.0) applied to the ASBR ranged from 0.8 to1.2 mg L^−1^ (half of the FA applied to the GSBR), which interfered with the performance of nitrite oxidising bacteria (NOB) because inhibition of NOB occurred at FA of 0.1 to 1 mg L^−1^ [28]. Therefore, the influent FA concentration was a plausible factor of nitrite accumulation in ASBR, conforming to the previous study [29].

The kinetic test for the ASBR was performed on day 135 (Fig. 4b), when the cycle started with 145 mg L^−1^ of TN and 83 mg L^−1^ were removed at the end of it (i.e., TN removal of 57%). The denitrification efficiency during the anoxic period was 18%, which was likely associated to the low biodegradability of tannic acid under anaerobic/anoxic conditions, whereas the ASBR was provided with mixing in the absence of aeration. Nonetheless, during the aerobic period, 68 mg L^−1^ of TN were removed via SND at an efficiency of 63%. Although this result was similar to the SND presented by the GSBR, the latter was loaded with an influent ammonium nitrogen concentration that was 1.5 times higher than GSBR.

For the same range of influent ammonium concentration (up to 200 mg L^−1^), the ANOVA results showed that the different performances of the GSBR and the ASBR regarding TN removal were statistically significant (*P*-value = 0.0004 at α =0.05).

#### Phosphorus removal (PR)

**Fig. 5:**
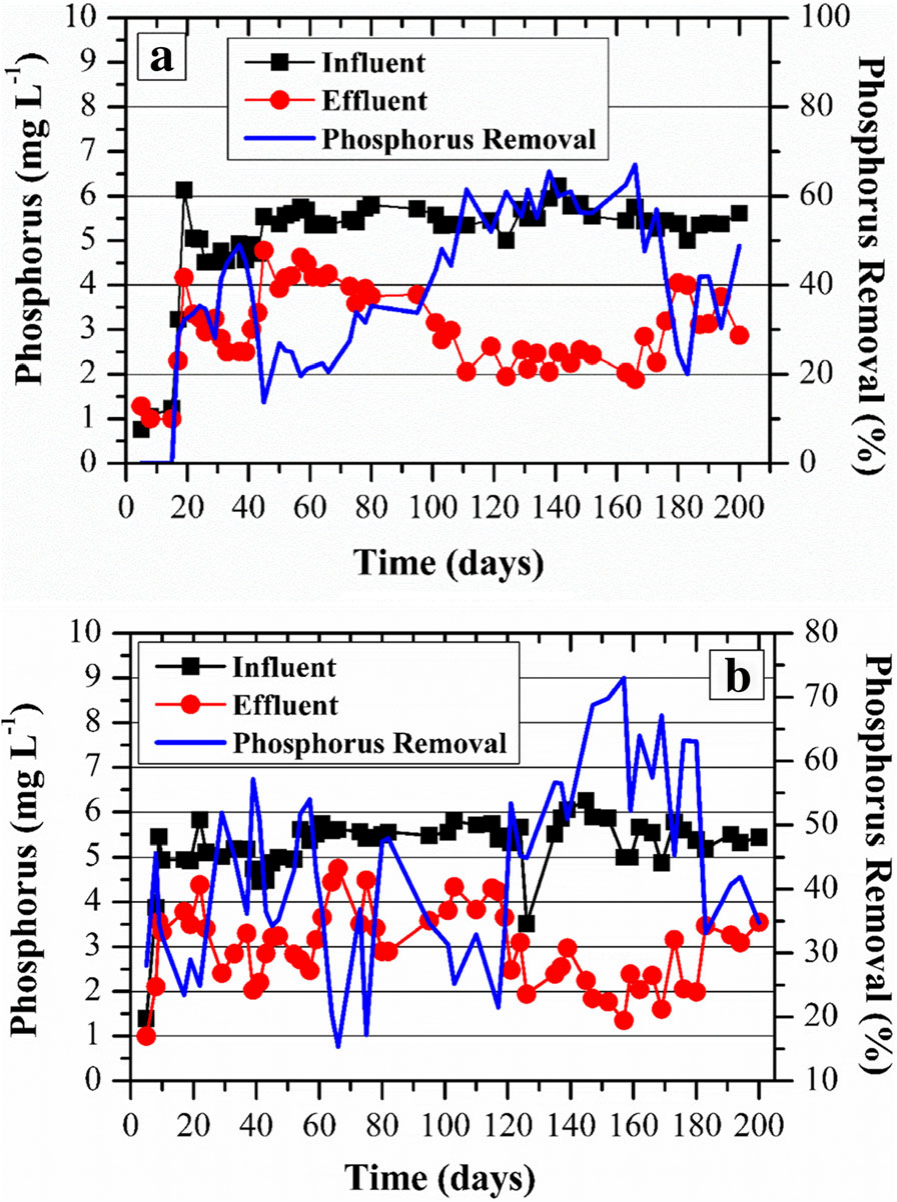
Phosphorus removal (PR) along with time for **a** the GSBR and **b** the ASBR

The influent phosphorus concentration of approximately 6 mg L^−1^ was found in both reactors (Fig. 5). However, phosphorus removal (PR) by the GSBR was gradually increased with time and reached a maximum efficiency of 54 ± 7% (Fig. 5a). The same trend was observed for the ASBR, which presented a maximum PR of 49 ± 14%. Despite these similar average efficiencies, it was clear that the ASBR presented considerable fluctuations in comparison with the GSBR, which were much more stable.

**Fig. 6:**
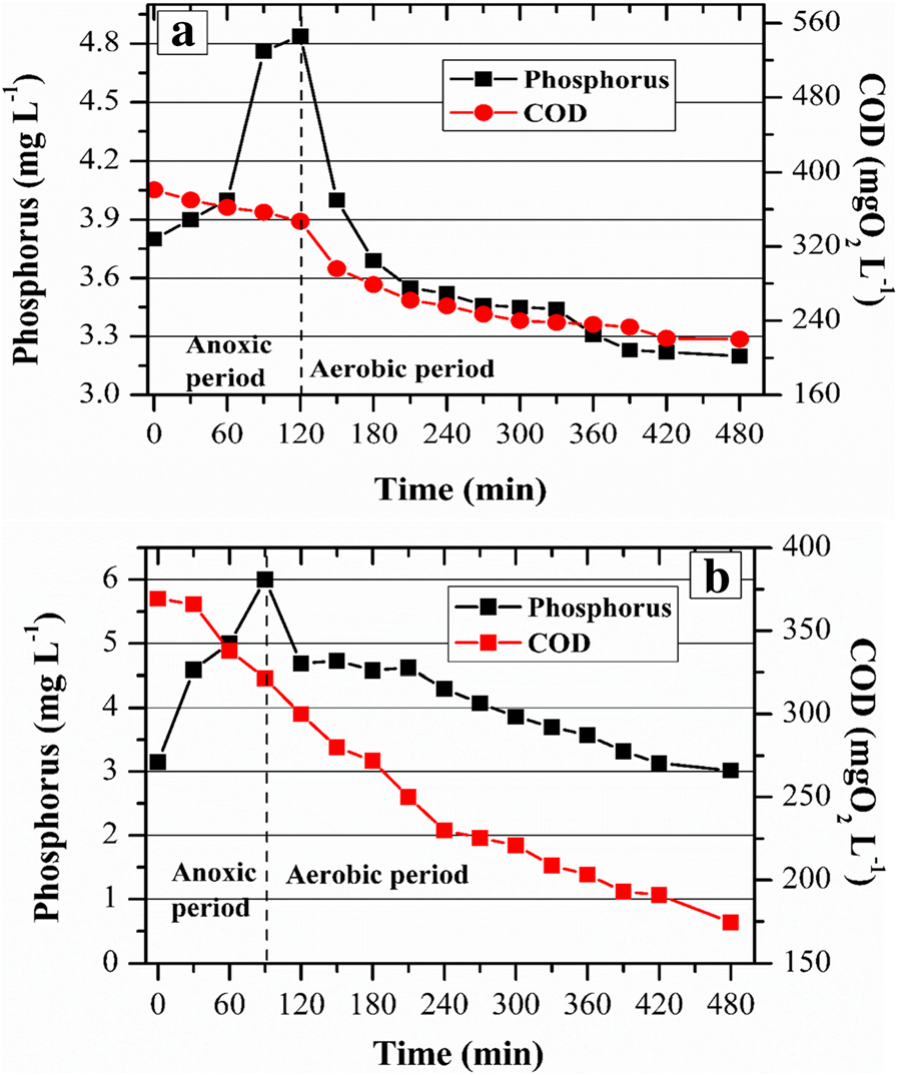
Phosphorus and COD removal during a typical cycle by **a** the GSBR and **b** the ASBR

As shown in Fig. 6a, a kinetic test was performed for the GSBR on day 95. During the anoxic period (first 90 min), phosphorus was released as COD was being consumed at a ratio P_released_/COD_consumed_ of 0.04. Afterward, phosphorus was assimilated by the PAO during the aerobic phase, and its removal at the end of the cycle was 34%. The kinetic test for the ASBR that was performed on the 135th day showed that the ratio P_released_/COD_consumed_ was 0.06 during the anoxic period. At the end of the aerobic phase, PR was 50% (Fig. 6b).

These moderate PR efficiencies could have been caused by the low biodegradability of tannic acid, which has been previously assessed [14, 16]. A previous study [30] reported that polyphosphate accumulating organisms (PAO) accounted for 70% of all bacteria from AGS when the ratio COD/P was 15:1, which was associated with a high phosphorus release/dissolved organic carbon uptake ratio (0.4). The opposite trend was observed when the COD/P ratio was 100:1, favoring the glycogen accumulating organisms (GAO), which compete with PAO for a carbon source. In the current research, the ratio COD/P throughout the experiment varied from 75:1 to 100:1, probably favoring the GAO activity. Additionally, phosphorus removal could have been affected by FNA. It was recently reported [31] that at an FNA (pH = 6.5) concentration of 1.2 μg L^−1^ inhibited 88% of PAO activity. In our study, FNA (pH = 6.5) varied from 1 to 5 μg L^−1^ for the GSBR, whereas it was extremely high for the ASBR, ranging from 13 to 97 μg L^−1^.

For the same range of influent ammonium concentration (up to 200 mg L^−1^), the ANOVA results showed that the differences among the performances of the GSBR and the ASBR regarding PR were statistically insignificant (*P*-value = 0.04 and α = 0.05).

## Conclusions

This study compared the performances of a GSBR and an ASBR in the treatment of old leachate containing different ammonium concentrations. From our results, it was concluded that the GSBR was much more efficient than the ASBR regarding the organic matter and nitrogen removal. The phosphorus removal efficiency was similar for both reactors. The granular biomass could tolerate influent ammonium concentrations 1.5 times higher than those applied to the ASBR. Although the GSBR was exposed to higher FA concentrations than the ASBR, no nitrite accumulation was observed. Further investigations should be addressed, especially with a focus on improving SND and phosphorus removal efficiencies; however, the use of AGS should be encouraged for a high-strength wastewater such as old landfill leachate.

## Abbreviations

AGS: Aerobic granular sludge
AOPs: Advanced oxidation processes
ASBR: Activated sludge sequencing batch reactor
BOD: Biological oxygen demand
COD: Chemical oxygen demand
DE: Denitrification efficiency
EPS: Extracellular polymeric substances
FA: Free-ammonia
FNA: Free-nitrous acid
GSBR: Granular sludge sequencing batch reactor
MLSS: Mixed liquor suspended solids
MLVSS: Mixed liquor volatile suspended solids
MSW: Municipal solid waste
NAR: Nitrite accumulation rate
NOB: Nitrite oxidizing bacteria
PAO: Poly-phosphate accumulating organisms
PN: Protein
PR: Phosphorus removal
PS: Polysaccharide
rbCOD: Ready biodegradable chemical oxygen demand
SBR: Sequencing batch reactor
sCOD: Soluble chemical oxygen demand
SRT: Sludge retention time
SVI: Sludge volum index
TAN: Total amminiacal nitrogen
TN: Total nitrogen
TP: Total phosphorus
VFA: Volatile fatty acid
